# Efficacy and outcomes of a highland prehospital trauma response team

**DOI:** 10.1177/00369330241277895

**Published:** 2024-09-05

**Authors:** Reuben Burgess, Tom Mallinson, Luke Regan

**Affiliations:** 1 NHS Grampian Foresterhill Health Campus, Aberdeen, Aberdeenshire, UK; 2 PICT Team, Emergency Department, Raigmore Hospital, Inverness, Highland, UK

**Keywords:** Scotland, trauma, highland, rural, prehospital

## Abstract

**Background and aims:**

The Scottish Highlands face unique prehospital care challenges due to population dispersity, mountainous terrain, seasonal weather, and higher trauma burden compared to the nearest Major Trauma Centres (MTCs) as highlighted by the Scottish Trauma Audit Group (STAG). Primary road/air transfer from scene to nearest designated MTC averages 1–5 hours, making prompt and informed utilisation of prehospital and in-hospital resources within the Highlands critical – comparative to other UK metropolitan regions where the trauma population majority lay within 20–45 minute transfer windows. This paper reviews the Highland pre-hospital immediate care and trauma (PICT) Team's trauma response through a retrospective review of PICT patient report forms (PRFs).

**Methods and results:**

The analysis highlighted increased trauma response by the team in the nature of attended callouts and interventions utilised. Improving trends of patient outcomes, increased advanced analgesia and medico-surgical intervention utilisation, and relative increase of road traffic collision attendance and trauma-specific calls were noted

**Conclusion:**

Results highlight the Scottish Highlands’ trauma burden and PICT's added value; with increased trauma response and improving outcomes. Despite the rate and ratio of major trauma not reducing PICT Team utilisation has, potentially led to fewer patients over narrower geography at later stages in emergency calls accessing the enhanced care doctor and advanced physician team than was achieved previously.

## Introduction

The Highland patient population, scattered as they are across rugged terrain and frequently subject to severe weather conditions, combined with the Scottish Trauma Audit Group (STAG)-reported prevalence of moderate and serious trauma in areas far from the nearest Major Trauma Centres (Edinburgh, Glasgow, or Aberdeen), presents a formidable challenge in prehospital response to traumatic injuries in the Highlands.^1,2^ Previous national trauma outcome inquiries have highlighted that improved trauma responses occur in organisations with trauma management system integration, regional hospital designation for trauma, direct trauma centre admission, early consultant involvement in the initial trauma response and transfers, and increased prehospital responder exposure to trauma management.^3^

**Figure 1.**
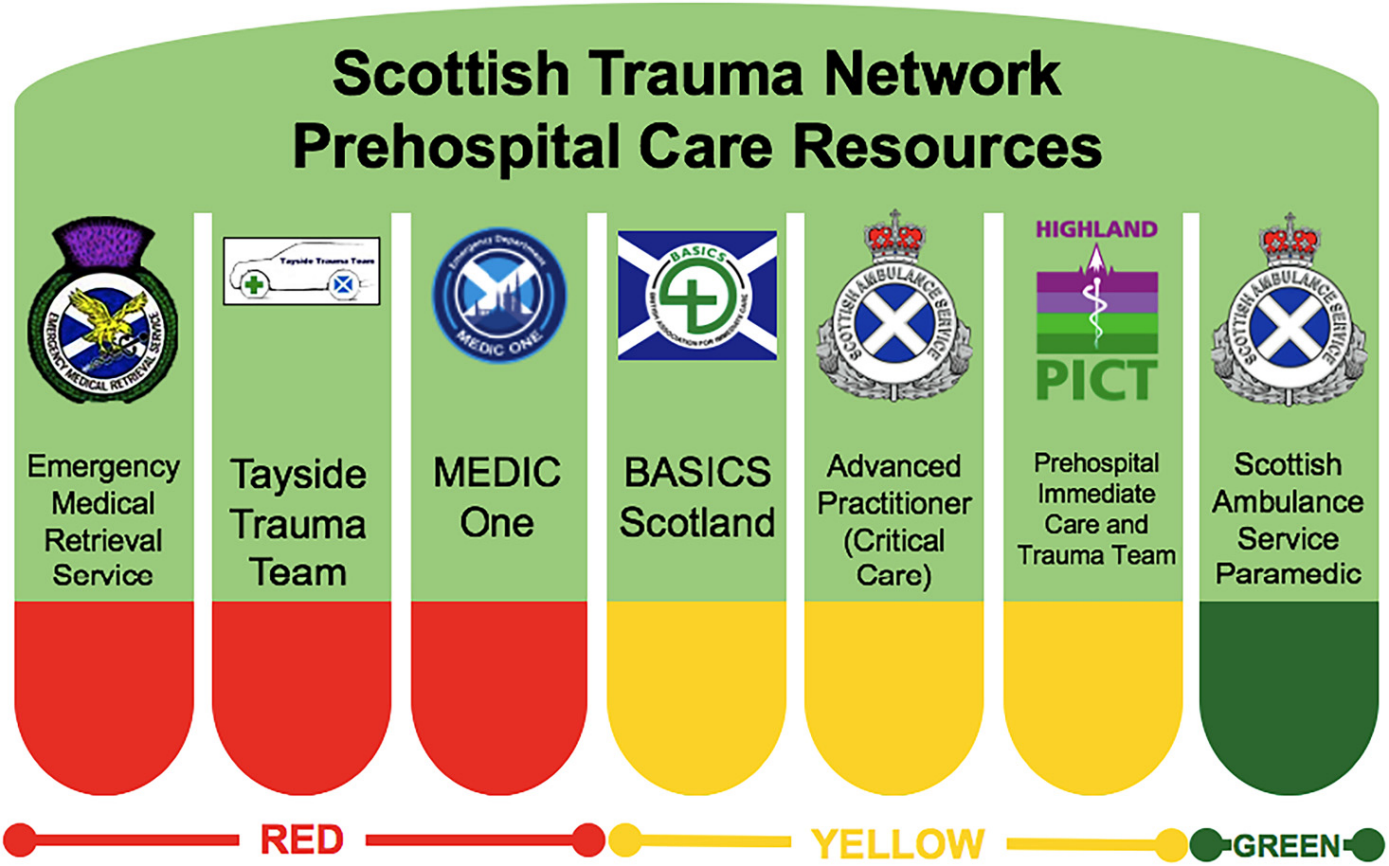
Red, yellow and green descriptors for Scottish prehospital resources.

The Scottish Trauma Network (STN) endeavours to address these challenges by integrating the resources of regional health boards, the Scottish Ambulance Service (SAS), and other prehospital services providing an enhanced level of care (e.g. BASICS Scotland, Tayside Trauma Team, or the Emergency Medical Retrieval Service).^4,5^ Across Scotland, strategic allocation of local resources and tasking to 999 calls based on resource capabilities allow ‘Green’ resources with comprehensive ambulance-based skillsets to collaborate with ‘Yellow’ and ‘Red’ resources (Figure 1), which provide an increasingly broad range of medico-surgical interventions, up to prehospital anaesthesia for ‘Red’ resources.^6,7^

**Figure 2.**
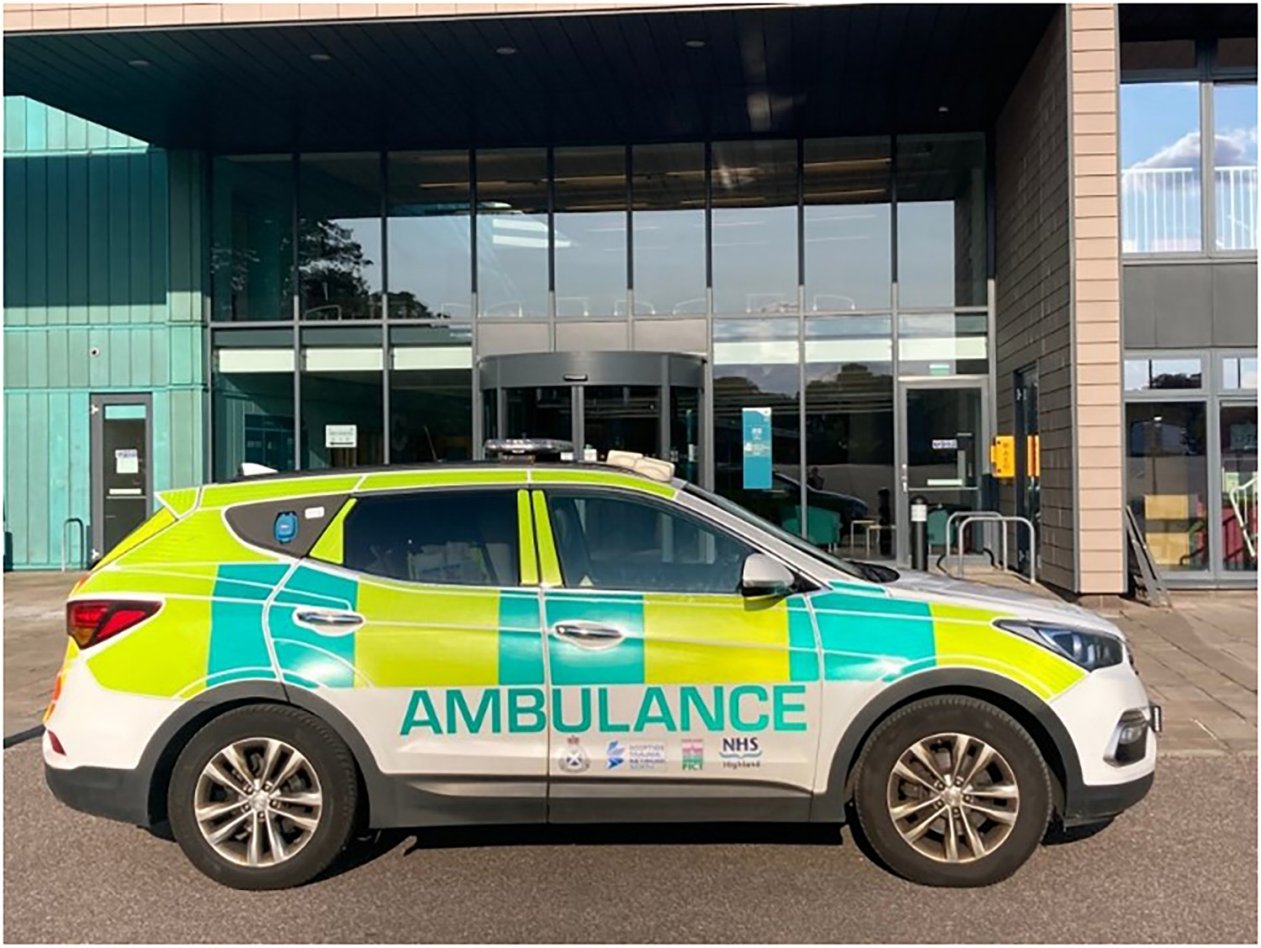
Highland prehospital immediate care and trauma (PICT) response car.

The Highland prehospital immediate care and trauma (PICT) Team serves as a ‘Yellow’ tier land-based trauma team in the Highlands operating out of the Raigmore Hospital Emergency Department (ED) in Inverness daily from 11:00 to 23:00.^7,8^ They are tasked to respond to emergency calls involving significant injury or critical illness and offer both remote support by advisory calls or on-scene assistance at the request of other services. The team is composed of an Advanced Practitioner in Critical Care (nurse or paramedic) and a senior doctor (Post-CCT or specialist grade), dispatched in a blue-light fast response vehicle (Figure 2) equipped with enhanced medico-surgical interventions such as capabilities to provide: amputation, regional anaesthesia, point-of-care ultrasound, sedation, mechanical cardiopulmonary resuscitation (CPR), and establishment of surgical airways.^7^ The senior doctors originate from diverse backgrounds including rural general practitioners (emergency/rural practitioners), retrieval specialists, intensive care consultants, anaesthetists and emergency medicine consultants. This provides a wide-ranging skillset within the cohort as a whole and encourages active peer learning within the team.^9^

This paper evaluates the work of the PICT team through the review of callout data of all-time service deployment to gain insight as to the potentially changing efficacy of the service in responding to trauma in the Scottish Highlands.

## Methods

A retrospective audit of PICT patient report form (PRF) data was undertaken, spanning 18 August 2021 to 18 March 2024 to assess call-out patterns, interventions employed and patient outcomes. Data transcribed into Microsoft Excel was segmented into 12-month periods spanning January to December of 2023, and 7-month intervals from 18 August to 18 March of 2021–22, 2022–23 and 2023–24. This was to allow for a direct comparison of time periods over as long a timeframe as possible. Duplicate entries were omitted and only dispositions for attended calls were analysed in detail. When absolute change was described, it was calculated by describing the numerical percentage change between timeframes (e.g. the percentage change in the number of trauma coded calls from 2022 to 2023) - this was pertinent in visualizing how these sections had a direct impact on workload or team resource. When relative change was described, it was calculated by finding the difference between the percentages a section made up in timeframes (e.g. the percentage of trauma coded calls in 2022 subtracted from the percentage of trauma coded calls in 2023) – this was pertinent in providing context to the changes in relation to, for example, numerical call volume.

## Results

Initial callout outcomes over a 12-month 2-year analysis of the 2022–2023 periods showed a decrease in total calls (−10.4%), overall number of attended, stood down, discharge and ward admittance, with an overall increase in ED admittance. This was mirrored in relative changes, respectively, barring attendance which showed a relative increase contrary to its overall decrease (Table 1). Outcomes over a 7-month 3-year analysis of the 2021–2022, 2022–2023 and 2023–2023 periods showed that scene discharge and ward admittance decreased in overall and relative volume between each year. After an initial decrease over the 2021/2022–2022/2023 period, stand-down rates both absolutely and relatively decreased over the 2022/2023–2023/2024 period. ED admittance relatively increased overall 7-month periods analysed (Table 2). Analysis of callout nature over a 12-month 2-year analysis showed medical and trauma-coded calls decreased and increased, respectively, in both absolute and relative change. Road traffic collision (RTC) coded calls decreased in absolute number, but relatively increased in volume. Non-RTC coded calls increased over the 2022–2023 period, with a decrease in coding for neck of femurs (NOFs) and fractures/breaks and an increase in dislocations, injuries and falls (Table 3).

**Table 1.** Annual initial callout analysis.

	12-month 2-year analysis of callouts			
	2022	2023	Absolute change	Relative change
Total calls	1420	1273	−10.4%	–
Attended	1151 (81.1%)	1056 (83%)	−8.3%	+1.9%
Stood down	269 (18.9%)	217 (17%)	−19.3%	−1.9%
Discharged at scene	373 (32.4%)	271 (25.7%)	−27.3%	−6.7%
Admitted directly to ward	154 (13.4%)	120 (11.4%)	−22.1%	−2%
Admitted to Emergency Department	559 (48.6%)	577 (54.6%)	+3.2%	+6%

**Table 2.** Temporal initial callout analysis.

7-month 3-year temporal analysis of callouts				
18 August 2021 to 18 March 2022				
Total calls	870			
Attended	763		87.7%	
Stood down	106		12.2%%	
Discharged at scene	277		36.3%	
Admitted directly to ward	91		11.93%	
Admitted to Emergency Department	353		46.26%	

18 August 2022 to 18 March 2023			Absolute change	Relative change
Total calls	717		−18%	–
Attended	590	82.29%	−22.7%	−5.41%
Stood down	127	17.7%	+19.8%	+5.5%
Discharged at scene	189	32%	−31.8%	−4.3%
Admitted directly to ward	70	11.86%	−23.1%	−0.07%
Admitted to Emergency Department	288	48.8%	−18.4%	+2.54%

18 August 2023 to 18 March 2024			Absolute change	Relative change
Total calls	675		−6%	
Attended	569	84.3%	−3.6%	+2.01%
Stood down	106	15.7%	−16.5%	−2%
Discharged at scene	123	21.62%	−34.9%	−10.38%
Admitted directly to ward	56	9.8%	−20%	−2.06%
Admitted to Emergency Department	337	59.23%	+17%	+10.43%

**Table 3.** Annual callout nature analysis.

	12-month 2-year analysis of callout nature			
	2022	2023	Absolute change	Relative change
Medical coded calls	739 (63%)	602 (56%)	−46.4%	−7%
Trauma coded calls	396 (34%)	400 (37%)	+1%	+3%
RTC's	164 (13.9%)	160 (15%)	−2.4%	+1.1%
Non-RTC-specified Musculoskeletal trauma	199 (16.9%)	208 (19.5%)	+4.5%	+2.6%
NOF	13 (1.1%)	10 (0.9%)	−23.1%	−0.2%
Fracture/break	52 (4.4%)	31 (2.9%)	−40.4%	−1.5%
Dislocation	8 (0.7%)	12 (1.1%)	+50%	+0.4%
Injury	18 (1.5%)	28 (2.6%)	+55.6%	+1.1%
Falls	108 (9.2%)	127 (11.9%)	+17.6%	+2.7%

RTC: road traffic collision; NOF: neck of femur.

Previous work has delineated PICT-specific interventions.^10,11^ These showed varying changes over a 12-month 2-year comparison. Among these was an increase in utilisation/deployment of point of care ultrasound (PoCUS), endotracheal tube (ETT), ketamine, fentanyl, adenosine and magnesium. There was a decrease in utilisation/deployment of mechanical CPR, intraosseous access, local anaesthetic use, atropine, steroids and antibiotics (Table 4).

**Table 4.** Annual medicoanalgesic utilisation analysis.

	12-month 2-year analysis of medico-analgesic utilisation			
	2022	2023	Absolute change	Relative change
PoCUS	6 (2.3%)	10 (4%)	(+67%)	+1.7%
Mech CPR	9 (3.5%)	7 (2.8%)	(−22%)	−0.7%
Endotracheal Intubation	16 (6.3%)	17 (6.7%)	(+6%)	+0.4%
IO access	29 (11.4%)	29 (11.5%)	–	−0.1%
Local Anaesthetics	30 (11.8%)	23 (9.1%)	(−23%)	−2.7%
Ketamine	24 (9.4%)	25 (9.9%)	(+4%)	+0.5%
Fentanyl	19 (7.5%)	31 (12.3%)	(+63%)	+4.8%
Diamorphine	0	2	–	–
Adenosine	2 (0.8%)	3 (0.8%)	(+50%)	–
Calcium	4 (1.6%)	4 (1.6%)	–	–
Magnesium	8 (3.1%)	10 (4%)	(+25%)	+0.9%
Atropine	14 (5.5%)	7 (2.8%)	(−50%)	−2.7%
Steroids	47 (18.4%)	41 (16.3%)	(−13%)	−2.1%
Antibiotics	47 (18.4%)	43 (17.1%)	(−9%)	−1.3%

IO: intraosseous; Mech CPR: mechanical cardiopulmonary resuscitation; PoCUS: point of care ultrasound.

Regarding end patient outcomes documented in PICT PRFs; intensive therapy unit (ITU) admittance, 30-day-death and 30-day-admittance reduced over all time periods analysed (Table 5).

**Table 5.** Temporal patient outcome analysis.

	Temporal analysis of patient outcomes documented in PRFs	
	2 -year 12-month comparison	
	1 January 2022 to 31 December 2022	1 January 2023 to 31 December 2023
Admitted to Intensive Care Unit	9	2
Died within 30 days	8	0
Admitted within 30 days	5	0

	3-year 7-month comparison		
	18 August 2021 to 18 March 2022	18 August 2022 to 18 March 2023	18 August 2023 to 18 March 2024
Admitted to Intensive Care Unit	8	1	0
Died within 30 days	8	1	0
Admitted within 30 days	2	0	0

PRF: patient report form

## Discussion

Absolute and relative analysis of annual and temporal changes in callouts highlight the decrease in total callout volume, with increasing relative trauma attendance. Increasing absolute and relative ED admittance and decreased ward admittance, discharge and stand-down rates may reflect more severe trauma directed to the PICT team.

While the total volume of RTCs has decreased, there is an increase in the relative number of RTCs attended. This may show a preference related to dispatching enhanced care to RTC-related trauma compared to other mechanisms. Non-RTC-specified musculoskeletal (MSK) trauma showed an increase which, upon breakdown, showed decreased NOF and fracture/breaks and increased dislocations, injuries and falls. This is likely to have resulted in fewer patients receiving prehospital nerve blocks for NOF or femur injuries. Data demonstrated that trauma-coded total calls, increased from 34% to 37%, with decreases in medical-coded calls, from 63% to 56%, depicting a large and increasing portion of trauma attended in the Highlands. This is mirrored by increasing RTC and non-RTC MSK trauma (13.9% to 15% and 16.9% to 19.5%, respectively). Changes in suggested PICT-specific medical interventions and pharmaceutical deployment^10,11^ varied. While enhanced analgesia use increased, other interventions expected to rise in serious trauma attendance, such as local anaesthetics and atropine decreased. This may be the result of changes in tasking criteria from the SAS. Irrespective of increasing trauma attendance PRF documented outcomes (ITU admittance, 30-day-mortality, and 30-day-admittance) all showed a decrease.

## Conclusion

The Highland PICT Team provides a land-based prehospital enhanced care service to a dispersed Scottish population. While the rate and ratio of major trauma have remained similar or increased, the utilisation of the PICT Team has reduced. This could lead to fewer patients over narrower geography and at later stages in emergency calls accessing the enhanced care doctor and advanced physician team than was achieved in previous years. Earlier and appropriate tasking to suitable calls is vital to allow their enhanced skillset to reach the patients who would benefit from these interventions. Future work could look at the selection of calls and the tasking and dispatch process for the team to better understand changes in tasking frequency. Early activation with subsequent stand down if not required could provide one route to increasing patient's access to the team's abilities.
